# HPLC-PDA Analysis of Polyacetylene Glucosides from *Launaea capitata* and Their Antibacterial and Antibiofilm Properties against *Klebsiella pneumoniae*

**DOI:** 10.3390/ph17091214

**Published:** 2024-09-15

**Authors:** Tariq M. Aljarba, Fatma M. Abdel Bar, Asmaa E. Sherif, Engy Elekhnawy, Galal Magdy, Reham M. Samra

**Affiliations:** 1 Department of Pharmacognosy, College of Pharmacy, Prince Sattam bin Abdulaziz University, Al-Kharj 11942, Saudi Arabia; t.aljarba@psau.edu.sa (T.M.A.); ae.sherif@psau.edu.sa (A.E.S.); 2 Department of Pharmacognosy, Faculty of Pharmacy, Mansoura University, Mansoura 35516, Egypt; rehamsamra@mans.edu.eg; 3 Pharmaceutical Microbiology Department, Faculty of Pharmacy, Tanta University, Tanta 31527, Egypt; engy.ali@pharm.tanta.edu.eg; 4 Pharmaceutical Analytical Chemistry Department, Faculty of Pharmacy, Kafrelsheikh University, Kafrelsheikh 33511, Egypt; galal_magdy@pharm.kfs.edu.eg; 5 Department of Pharmaceutical Analytical Chemistry, Faculty of Pharmacy, Mansoura National University, Gamasa 7731168, Egypt

**Keywords:** *Klebsiella pneumoniae*, bacterial virulence, biomarker quantification, HPLC-PDA, *Launaea capitata*, quorum sensing, biofilm formation, biofilm genes, bidensyneoside A, gymnasterkoreaside A

## Abstract

**Background/Objectives**: Bacterial resistance and virulence are challenges in treating bacterial infections, especially in *Klebsiella pneumoniae*. Plants of the *Launaea* Cass. genus are used traditionally to address a variety of diseases, including infections, but the potential bioactive compounds are unknown. Our goals were to verify the potential contribution of two major polyacetylene glycosides isolated from our previous study, (3*S*,6*E*,12*E*)-6,12-tetradecadiene-8,10-diyne-1-ol 3-*O*-*β*-D-glucopyranoside (**1**) and bidensyneoside A (syn. gymnasterkoreaside A) [(3*R*,8*E*)-3-hydroxy-8-decene-4,6-diyn-1-yl *β*-D-glucopyranoside] (**2**), to the anti-infective properties of *Launaea capitata* and to develop a dependable HPLC method for their quantification; **Methods**: On a panel of *K. pneumoniae* clinical isolates, the antibacterial action of **1**, **2**, and the methanol extract of the whole *L. capitata* plant were evaluated by broth microdilution assay, while their antibiofilm action was evaluated by the crystal violet assay. qRT-PCR investigated *lux*S, *mrk*A, *wzm*, and *wbb*m genes that encode biofilm formation and quorum sensing (QS). The antibacterial activity of **1** was revealed by employing mice infection. Chromatographic separation was conducted using isocratic elution on a Hypersil BDS C18 column using a photodiode array (PDA) detector; **Results**: Compound **1** showed antibacterial activity with MIC values of 16–128 µg/mL. It remarkably reduced strong and moderate biofilm-forming bacterial isolates from 84.21% to 42.1% compared with the extract (68.42%) and **2** (78.95%). Compound **1** also downregulated the QS genes, *lux*S, *mrk*A, *wzm*, and *wbb*m, and exhibited in vivo antibacterial action through the enhancement of the histological construction of the liver and spleen, decreased TNF-*α* immunoreaction, bacterial burden, and the inflammatory mediators IL-1*β* and IL-6. A successful HPLC-PDA approach was developed to separate the binary mixture of **1** and **2** in less than 10 min with high sensitivity, with detection limits down to 0.518 and 0.095 µg/mL for **1** and **2**, respectively; **Conclusions**: Compound **1** exhibited remarkable antibacterial and antibiofilm properties and may contribute to the anti-infectious traditional uses of *L. capitata*, meriting further clinical studies and serving as a reliable quality control biomarker for the plant.

## 1. Introduction

Bacterial resistance poses a serious and expanding danger to antibiotic efficacy, potentially undoing decades of advancements in medicine. Antibiotic resistance results from the ability of bacteria to adapt so they can avoid the effects of antimicrobial agents. The synthesis of enzymes that break down antibiotics, modifications to drug targets, or improved efflux pumps that remove medications from bacterial cells are some of the processes that might cause this phenomenon. The emergence of resistant strains, such as multidrug-resistant *Mycobacterium tuberculosis* and methicillin-resistant *Staphylococcus aureus* (MRSA), complicates treatment plans and raises the risk of longer sickness, greater medical expenses, and higher death rates. The selection pressure that promotes resistance is fueled by the overuse and abuse of antibiotics in agriculture and human health, which worsens the issue [1,2]. 

The Gram-negative *Klebsiella pneumoniae* bacterium is considered a common nosocomial pathogen. In addition, it frequently triggers severe community-acquired infections [3]. The diseases caused by such bacteria are numerous, including respiratory, urinary, and systemic infections [4]. *K. pneumoniae* possesses various virulence factors like a capsule, biofilm formation, and iron carriers [5]. Biofilms compromise bacterial cells that can propagate on living or non-living surfaces [6]. They are frequently immersed in a self-assembled polysaccharide matrix. The infections triggered by the biofilm-producing bacterial cells are considered a significant health problem due to the high ability of such bacterial isolates to withstand the antibacterial compounds and the human immune system [7,8].

Bacteria utilize the quorum-sensing (QS) system to communicate among themselves. Such communication has a significant role in controlling many biological functions, like biofilm formation, evolving antibiotic resistance, and the secretion of various extracellular enzymes [9,10]. *K. pneumoniae* employs the LuxS/AI-2 as a self-induction factor for employing QS. This system in *K. pneumoniae* bacteria can regulate and control bacterial biofilm formation [11,12]. Therefore, hindering QS could assist us in the battle against these pathogenic bacteria. It was reported that the bacterial phenotype in biofilms is not as that of the planktonic cultures. This is attributed to the multiple bacterial interactions in the formed biofilm [13]. The cells embedded in the biofilm have a greater frequency of mutating and a higher rate of transfer of the genetic materials in comparison with the planktonic cells. Consequently, bacterial cells in biofilms could be up to 1000 times more antibiotic-resistant than their planktonic counterparts [14]. In addition to the *K. pneumonia’*s virulence factors, their isolates extensively resist numerous antibiotics. Hence, many studies are interested in finding novel therapeutic alternatives to current antibiotics [15,16]. 

Traditional medicines have utilized plants for treating and managing various disorders since the dawn of human civilization. Various species within the Asteraceae family have the potential for development as bacteriostatic agents and for combating multidrug-resistant bacteria, such as *Artemisia*, *Baccharis*, and *Vernonia* spp. [17]. Plants of the genus *Launaea* (Asteraceae) are well-adapted to arid and semiarid environments, with a notable presence in the Arabian Peninsula, North Africa, and Southwest Asia [18,19,20]. Because of its nutritional value and health advantages, native people in these nations employ *Launaea* spp. as edible greens [21,22]. Traditionally, numerous plants in this genus have been employed for a variety of purposes, including bitter stomachics, diarrhea and gastrointestinal issues, anti-inflammatory agents, skin conditions and infected wounds, liver complaints, children’s fevers, soporifics, galactagogues, diuretics, and insecticides [23]. The plants of the *Launaea* genus are rich in bioactive compounds, such as pentacyclic triterpenes (e.g., oleanane, lupane, ursane, and gammacerane derivatives), phenolic compounds (e.g., benzoic and cinnamic acids, lignans, coumarins, and flavonoids), galactolipids, and polyacetylenes [22,24,25,26,27].

On the other hand, the most popular chromatographic techniques used in the literature to analyze polyacetylenes are capillary gas chromatography with flame ionization or mass detectors (GC-FID or GC-MS) [28,29,30] and high-performance liquid chromatography with UV or mass detectors (HPLC-UV or LC-MS) [31,32,33]. 

**Figure 1:**
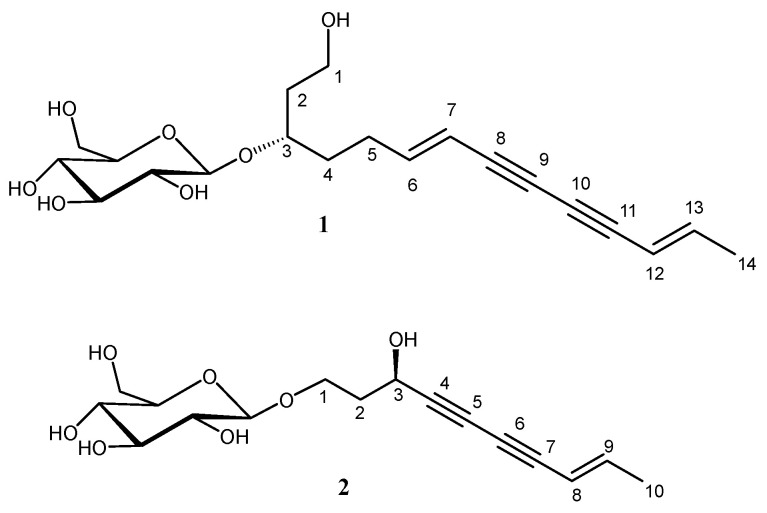
Structures of the studied polyacetylene glycosides, (3*S*,6*E*,12*E*)-6,12-tetradecadiene-8,10-diyne-1-ol 3-*O*-*β*-D-glucopyranoside (**1**) and bidensyneoside A (syn. gymnasterkoreaside A) [(3*R*,8*E*)-3-hydroxy-8-decene-4,6-diyn-1-yl *β*-D-glucopyranoside] (**2**).

The ethnopharmacological significance of the *Launaea* genus stems from its traditional use in treating infections and fevers, reflecting its historical role in addressing health issues such as child fever and infected wounds [20,23]. Moreover, some polyacetylenes from *L. capitata* were shown to have in vitro antibacterial and anti-biofilm activities against *Staphylococcus aureus* [24]. In light of these studies as well as further reports describing the significance of polyacetylene glycosides as antimicrobial agents [34], new studies on other bacteria are required to understand whether polyacetylene glycosides contribute to the traditional medicinal use of *L. capitata*. Herein, we aimed to elucidate the credible antibacterial, antibiofilm, and anti-quorum-sensing actions of the methanol extract of the whole *L. capitata* plant alongside two isolated polyacetylene glycosides, **1** and **2** (Figure 1), on *K. pneumoniae* clinical isolates by implementing in vitro assays and an experimental animal model; also, to employ an optimized technique utilizing an HPLC method with a photodiode array detector (HPLC-PDA) to detect and quantify the designated polyacetylene glycosides (**1** and **2**) as reliable biomarkers and for the analysis and quality control procedure of *L. capitata* total methanol extract.

## 2. Results

### 2.1. In Vitro Antimicrobial Action

#### 2.1.1. Susceptibility Assay

**Figure 2:**
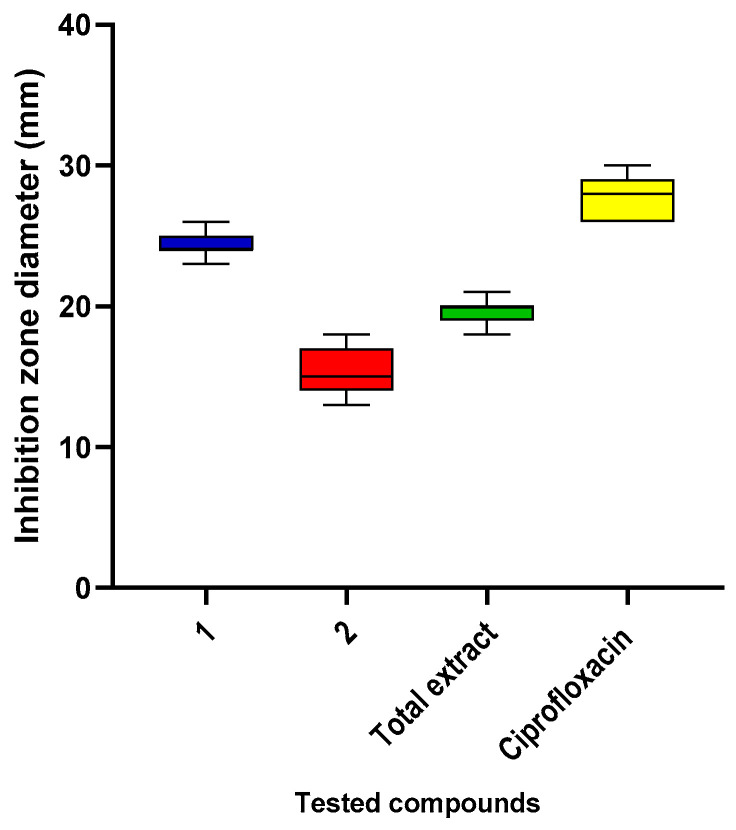
Inhibition zone diameters of the total extract, **1**, **2**, and ciprofloxacin.

Using the agar diffusion experiment, we investigated the sensitivity of *K. pneumoniae* isolates to the methanol extract of the entire *L. capitata* plant, in addition to compound **1**, compound **2**, and ciprofloxacin. As revealed in Figure 2, the inhibition zone diameters of **1** and **2** are recorded as a preliminary investigation of the antibacterial activity of the tested compounds. Then, the MICs were measured (Table 1).

#### 2.1.2. Antibiofilm Activity

The antibiofilm actions of the total extract, **1**, and **2** were recorded using the crystal violet test. Remarkably, **1** exhibited a higher antibiofilm action than the total extract and **2**, as the percentage of *K. pneumoniae* isolates that form a biofilm in a strong and moderate manner decreased from 84.21% to 42.1%, 78.95%, and 68.42% after treatment with **1**, **2**, and the total extract, respectively (Table 2).

#### 2.1.3. Quantitative Real-Time PCR (qRT-PCR)

**Figure 3:**
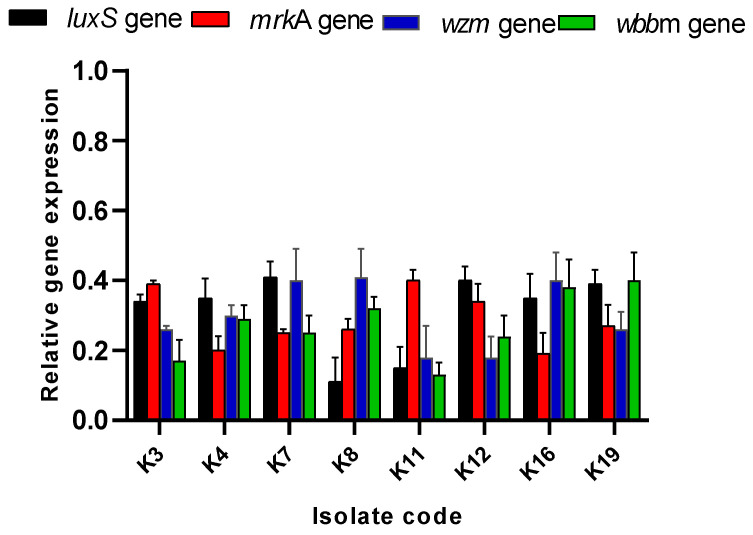
Impact of compound **1** on the QS and biofilm gene expression.

Owing to the antibiofilm action of **1** revealed by the crystal violet assay, qRT-PCR was employed to unveil its consequence on the expression of the QS and biofilm-related genes. A downregulation of the genes was observed after treatment with **1** (Figure 3).

### 2.2. In Vivo Assay

Owing to the potent in vitro antibacterial action of **1** on *K. pneumoniae*, we tested its effectiveness in vivo using a mice model of systemic infection.

#### 2.2.1. Bacterial Count (Tissue Burden) 

**Figure 4:**
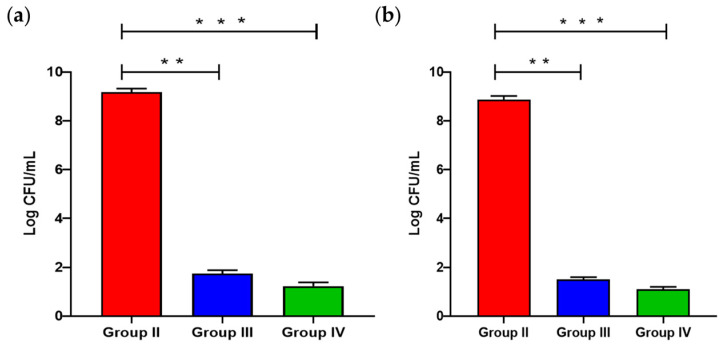
Burden in (**a**) liver and (**b**) spleen of the mice groups. Groups II–IV received injections of *K. pneumoniae* and were orally given 0.9% normal saline daily (Group II), compound **1** (Group III), and ciprofloxacin (Group IV). The two asterisks indicate an extensive variation (*p* < 0.05) between groups II and III. The three asterisks indicate an extensive variation (*p* < 0.05) between groups II and IV.

The count of *K. pneumoniae* in the tissues was evaluated to disclose the influence of **1** on the bacterial load within the examined tissues. As shown in Figure 4, Group III (the group treated with compound **1**) exhibited a substantial decline in the count (*p* < 0.05) of *K. pneumoniae* in comparison with the infected group.

#### 2.2.2. Histopathology

**Figure 5:**
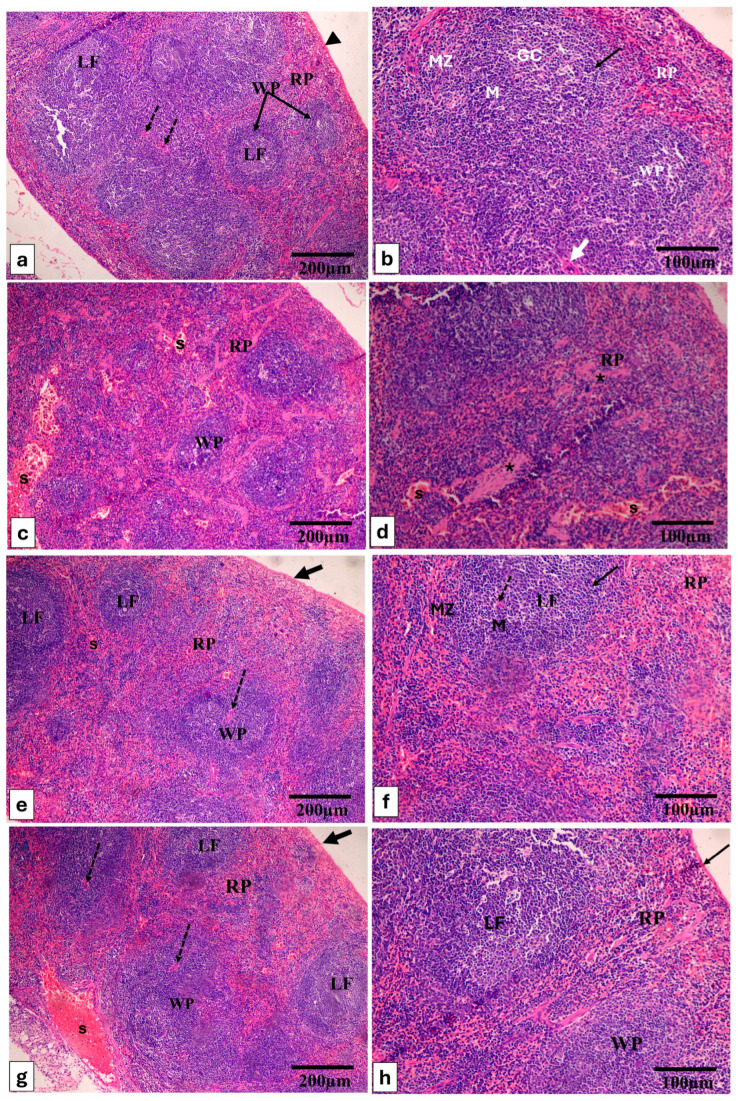
H&E-stained sections of spleen of (**a**,**b**) Group (I) displays the typical characteristics of both white pulp (WP) and red pulp (RP) encapsulated within a capsule (indicated by black arrowhead). The WP comprises the central arteriole (white arrow), lymphoid follicles (LF), featuring germinal centers (GC), and a mantle region (M), all situated within a loosely organized marginal zone (MZ). The RP is characterized by the presence of lymphocytes, trabeculae, and sinusoids. (**c**,**d**) In Group (II), there is a notable deviation from normal structure, with a contracted white pulp (WP) and an expanded red pulp (RP). The red pulp exhibits congested, dilated splenic sinuses (S). Numerous cells in the white pulp (WP) appear vacuolated, and prominent fibrous trabeculae are evident (marked by an asterisk). (**e**,**f**) Group (III) shows a splenic architecture that is almost typical, with both white pulp (WP) and red pulp (RP) presenting near-normal configurations; (**g**,**h**) Group (IV) displays an approximately regular splenic construction, with a dilated, congested splenic sinus. H&E × 100, scale bar = 200 μm, H&E × 200, scale bar = 100 μm.

**Figure 6:**
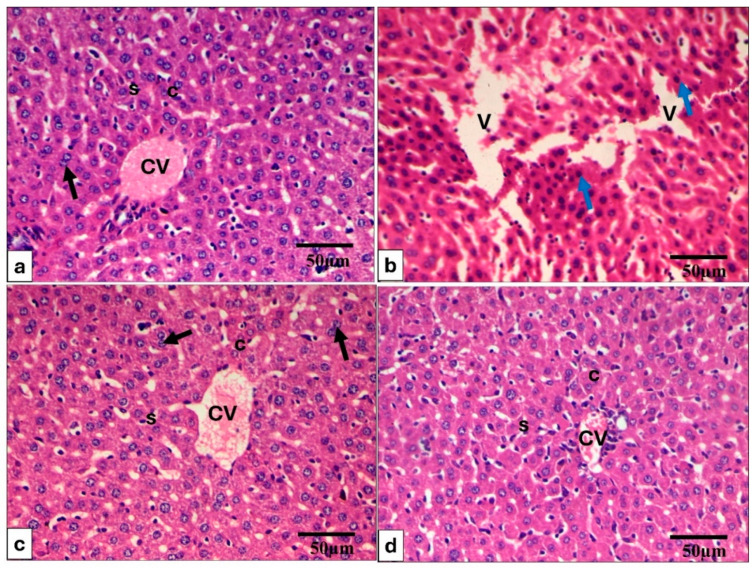
H&E-stained hepatic sections. (**a**) In Group (I), the liver exhibits typical histological features, including well-organized hepatic cords (C) extending from the central vein (CV) and disjointed by hepatic sinusoids (S). The peri-central and midzone hepatocytes are characterized by polyhedral shapes, round vesicular nuclei, and granular eosinophilic cytoplasm (indicated by a black arrow). The sinusoids (S) are lined with endothelial cells and interspersed Kupffer cells. (**b**) Group (II) displays an absence of the normal hepatic architecture, compacted hepatic sinusoids, numerous pyknotic nuclei (blue arrow), and diffuse vacuolar degeneration of hepatocytes which exhibit numerous vacuoles (V) and ballooning; (**c**,**d**) Groups (III and IV) display a marked improvement and regaining of the normal hepatic structure. H&E × 400, scale bar = 50 μm.

The H&E-stained spleen and liver are shown in Figure 5 and Figure 6.

#### 2.2.3. Immunohistochemistry

**Figure 7:**
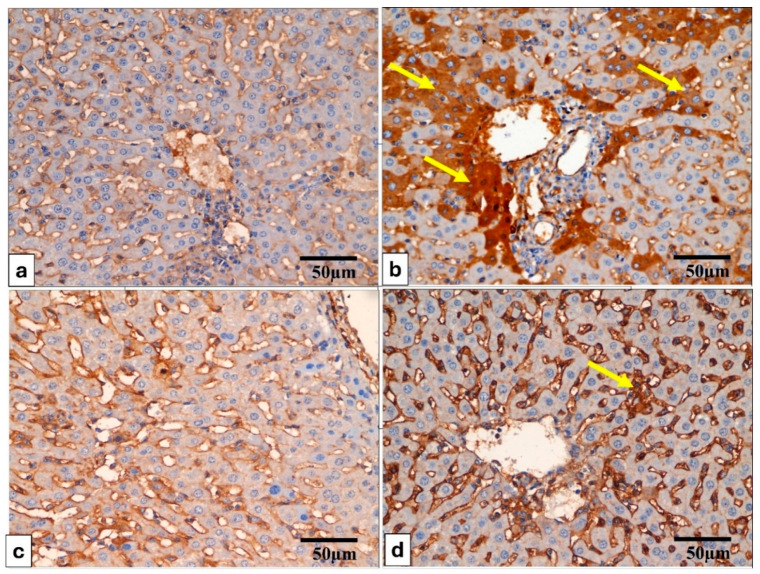
Liver sections stained for TNF-*α* reveal the following: (**a**) in Group (I), TNF-*α* is absent in the cytoplasm of hepatocytes. (**b**) Group (II) shows pronounced TNF-*α* positivity, evidenced by a distinct brown staining in the cytoplasm of hepatocytes (indicated by yellow arrows). (**c**,**d**) Groups (III and IV) display a sparse presence of TNF-*α*-positive cells (yellow arrow), with the majority of cells showing no reaction. TNF-*α* × 400, scale bar = 50 μm.

**Figure 8:**
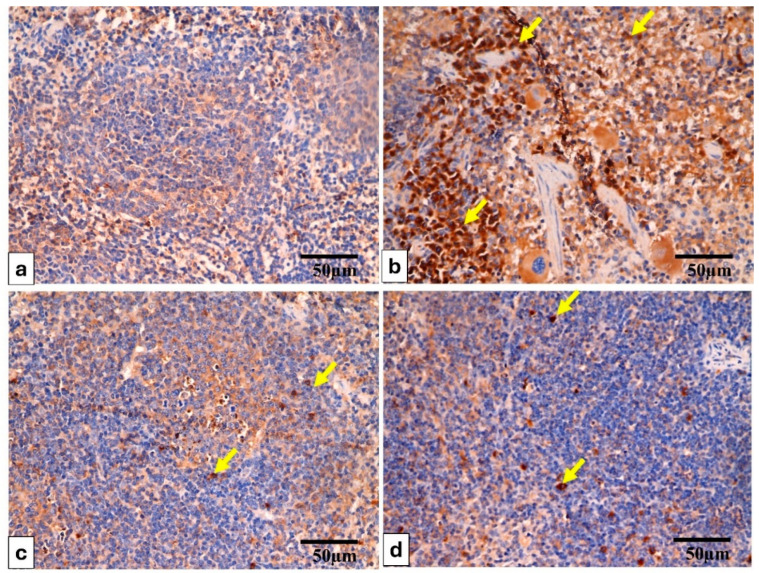
Sections of the spleen stained for TNF-*α* reveal the following: (**a**) in Group (I), there is an absence of TNF-*α* within the cytoplasm of the cells. (**b**) Group (II) demonstrates intense TNF-*α* positivity, with prominent brown staining evident in the cytoplasm of the majority of cells (indicated by yellow arrows). (**c**,**d**) Groups (III and IV) show a limited number of TNF-*α*-positive cells (yellow arrow), with many cells exhibiting no detectable TNF-*α* staining. TNF-*α* × 400, scale bar = 50 μm.

The tumor necrosis factor alpha (TNF-*α*) immunostained liver and spleen are shown in Figure 7 and Figure 8.

#### 2.2.4. Enzyme-Linked Immunosorbent Assay (ELISA)

**Figure 9:**
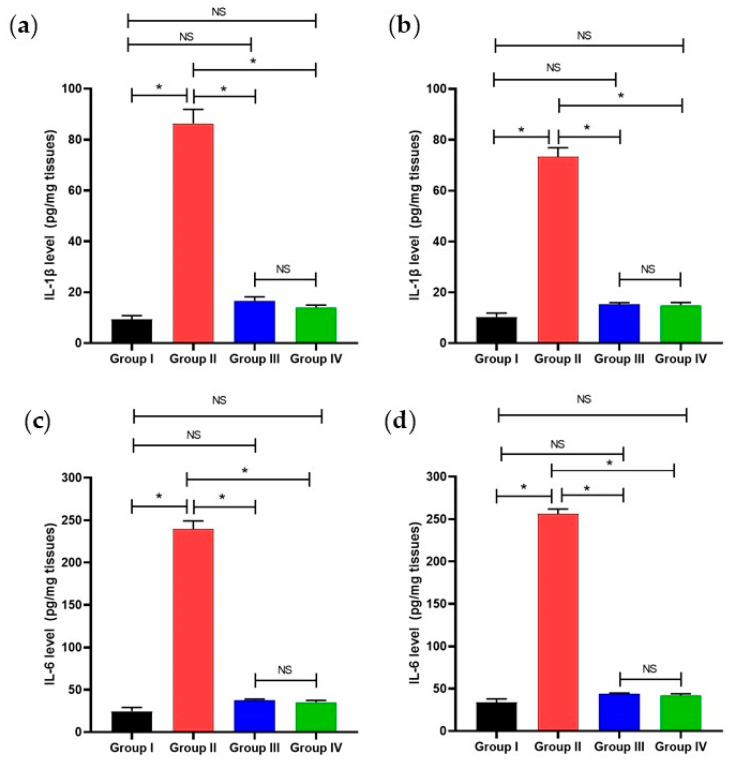
Bar charts represent the levels of IL-1*β* as well as IL-6 in the liver (**a**,**c**) and spleen (**b**,**d**). The single asterisk indicates a considerable variation (*p* < 0.05). The (NS) indicates a non-considerable variation (*p* > 0.05).

The levels of IL-1*β* as well as IL-6 were recorded in the tissues of the experimental groups employing ELISA, as presented in Figure 9.

### 2.3. HPLC-PDA Analysis

**Figure 10:**
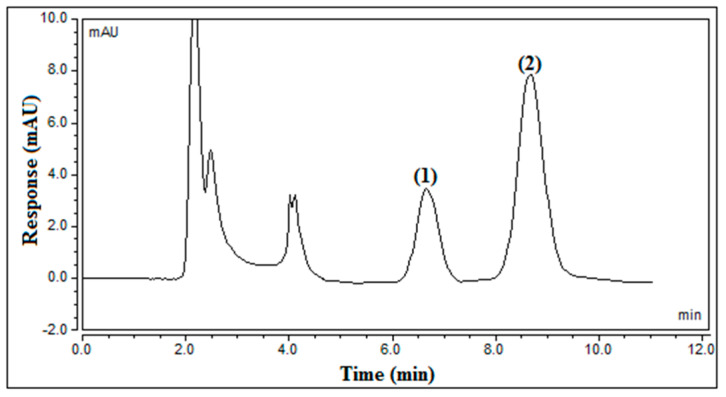
Typical chromatograms of compounds (**1**) and (**2**) in the total methanolic extract of the entire *Launaea capitata* plant under the optimal chromatographic conditions.

The quantitative analysis of the two biomarkers (**1** and **2**) was efficiently achieved using a simple HPLC-PDA method, which was carefully optimized and developed. The studied experimental parameters included the type and ratio of the organic modifier, the type of column, the flow rate, and the detection wavelength. Acetonitrile and methanol were tested, and methanol was the optimal organic modifier, resulting in the optimal resolution in a short run time (less than 10 min), while acetonitrile resulted in overlapped peaks. Afterward, the ratio of methanol to double dist. H_2_O was examined in the range of 20.0–50.0%, and the best separation was achieved when the ratio of methanol was set to 50.0%. Two columns were tested, and the Hypersil BDS C18 column (5 μm particle size, 250 mm × 4.6 mm i.d.) resulted in the best separation and resolution. The flow rate was also investigated over the range of 0.8–1.5 mL/min, and 1.0 mL/min was the optimal flow rate, in terms of the method’s sensitivity and resolution. The UV spectrum of each biomarker was recorded, and the optimal PDA detection wavelength was found to be 284 nm, producing the highest sensitivity for the two biomarkers. Accordingly, the optimal chromatographic conditions for the developed HPLC-PDA method were as follows: the two biomarkers were analyzed using an RP-C18 column (Hypersil BDS, 5 μm particle size, 250 mm × 4.6 mm i.d.) with a mobile phase consisting of a 50:50% *v*/*v* mixture of methanol and double H_2_O. Isocratic elution was performed at a flow rate of 1.0 mL/min with an injection volume of 30.0 μL. The PDA detector was set at 284 nm to examine and measure the studied compounds. Before the examination, it was crucial to condition the column for 20 min. Figure 10 shows the typical chromatogram of the two biomarkers in the total methanolic extract under the optimal chromatographic conditions, where the retention time was 6.78 min and 8.07 min for **1** and **2**, respectively.

**Figure 11:**
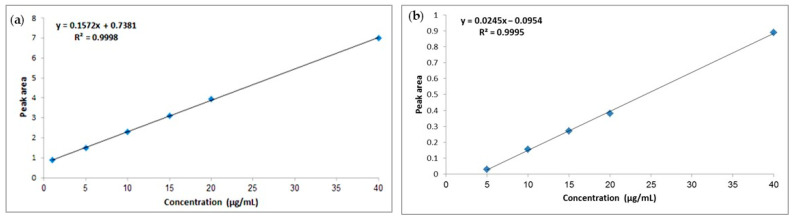
Calibration graphs for the HPLC-PDA determination of **1** (**a**) and **2** (**b**).

The quantitation of the two analytes was performed using the corresponding regression equations produced from the calibration curves constructed using the pure form of each compound in the ranges of 5.0–40.0 µg/mL and 1.0–40.0 µg/mL for **1** and **2**, respectively (Figure 11). 

The total extract was injected under the optimal chromatographic conditions and the peak area for each compound was obtained; then, the concentration of each compound was as follows: 

Compound **1**:

Regression Equation: Y = 0.0245x − 0.0954 The concentration of compound **1** in the total extract = 29.89 µg/mL (1)

Compound **2**:

Regression Equation: Y = 0.1572x + 0.7381 The concentration of compound **2** in the total extract = 32.887 µg/mL (2)

Additionally, the analytical performance data for the developed HPLC method were studied according to ICH guidelines [35] and summarized in Table 3.

The proposed method revealed high % recoveries for both compounds, as demonstrated in Table 4.

## 3. Discussion

*K. pneumoniae* is abundant in various environments and normal flora on human mucosal surfaces. However, it can trigger many infections, which could be very severe and life-threatening [36]. Moreover, such pathogens resist numerous antibiotics, especially in clinical settings [37,38,39]. As plants are wealthy sources of various bioactive compounds [40], we explored the susceptibility of *K. pneumoniae* isolates to the methanol extract of the entire *L. capitata* plant beside compounds **1** and **2** by the agar diffusion assay. This method could be preliminary to reveal whether the investigated extract or compounds have antibacterial activity [41] by detecting the diameter of the inhibition zone around the wells. Thus, we used the broth microdilution method to detect the values of the MICs of the investigated samples. The MIC values of the tested extract, which varied from 512 to 1024 µg/mL, indicated moderate antibacterial activity. Regarding the tested compounds, the MICs of **2** ranged from 1024 to 2048 µg/mL, while **1** showed better antibacterial activity, as revealed by the MIC range from 16 to 128 µg/mL. Previous studies have demonstrated potential antimicrobial activities for various polyacetylenes [42,43]. For instance, Jeong et al. (2010) demonstrated that (6*E*,12*E*)-tetradecadiene-8,10-diyne-1,3-diol, a hydrolysis product derived from a 1,3-diacetate derivative extracted from the roots of *Atractylodes japonica,* has shown significant antibacterial efficacy against MRSA [44]. However, Jeong et al. (2010) may misassign the name and/or structural drawing of their compounds (i.e., the free and the 1,3-diacetate derivatives) by giving a C_14_-carbon chain for both of them [44]. This is because the chemical structure referring to diacetyl-atractylodiol was established in the literature by several reports from the same plant (i.e., *A. japonica*) as (5*E*,11*E*)-tridecadiene-7,9-diyne-1,3-diol, diacetate with a C_13_-carbon chain [45,46,47]. Still, the results obtained by Jeong et al. (2010) support our antimicrobial research findings, which confirmed the significant antibacterial efficacy of polyacetylene derivatives.

Biofilm is a critical virulence factor for many bacteria, enabling the bacterial cells to exchange resistance genes [48]. It was reported that most chronic and recurrent infections, like endocarditis and urinary tract infections, are frequently linked to the capacity of the causative pathogen to customize biofilm [49]. *K. pneumoniae*, particularly, can form biofilms on indwelling devices which predisposes invasive and life-threatening infections [50]. Herein, we elucidated the potential antibiofilm action of the methanol extract of *L. capitata* alongside two polyacetylene glycosides (**1** and **2**) isolated from this plant. The percentage of strong/moderate *K. pneumoniae* biofilm-forming isolates decreased from 84.21% to 68.42% after treatment with *L. capitata* extract. Compound **1** exhibited an efficient antibiofilm action as it diminished the investigated bacterial isolates from 84.21% to 42.1%. Thus, qRT-PCR was used to study the antibiofilm action at the gene expression level. QS controls and organizes biofilms by regulating an essential step in biofilm formation (adherence to biotic and abiotic surfaces) [51]. Here, **1** has downregulated the genes encoding QS and biofilm. Therefore, we investigated the antibacterial action of **1** in an animal model to study its effectiveness in vivo. Remarkably, the compound **1**-treated group has shown a noticeable improvement and regaining of the normal hepatic and splenic structures compared with the infected group. The inflammatory markers that usually increase in the animal body in response to the invading pathogen [52] were remarkably decreased in the compound **1**-treated group. The intensities of inflammatory indicators, like IL-1*β* [53], IL-6 [54], and TNF-*α* [55], usually rise during bacterial infections as they are part of bacterial pathogenesis [56].

The structural features of compound **1** as a polyacetylene glycoside are mainly responsible for the evident antibacterial activity. Chemically, compound **1** is classified as a polyacetylene glycoside. Polyacetylene glycosides are a group of natural bioactive compounds bearing a sugar moiety and an aglycone part. The aglycone part contains two to several acetylenic (triple) bonds. Polyacetylenes have been reported to have various pharmacological activities like immunomodulation [57], anti-inflammatory [58,59], and hepatoprotective potentials [60]. The diverse biological activities of the aglycone part were mainly attributed to the presence of the reactive acetylenic bonds as they exert their biological actions through interacting with cellular proteins, hence promoting diverse biological effects, such as anticancer, antifungal, antibacterial, and anti-inflammatory effects [31]. For instance, bidensyneoside A1 (**2**) from *L. capitata* was reported to have in vitro antibacterial and anti-biofilm activity against *S. aureus* [24]. Our results indicated that compound **1**, namely (3*S*,6*E*,12*E*)-6,12-tetradecadiene-8,10-diyne-1-ol 3-*O*-*β*-D-glucopyranoside, showed better antibacterial and antibiofilm actions compared with bidensyneoside A1 (**2**).

Accurate quantification of the two biomarkers (**1** and **2**) was accomplished through a simple HPLC-PDA technique that was meticulously developed and optimized. The results obtained for biomarkers **1** and **2** revealed interesting insights into their concentrations within the total extract. The concentration of **1** in the total methanol extract of *L. capitata* was determined to be 29.89 µg/mL. On the other hand, biomarker **2** was found to be present at 32.887 µg/mL, demonstrating a concentration that was nearly equivalent to that of **1**. These results signify the abundance of **1** and **2** within the total plant extract in appreciable concentrations. The results could provide valuable information regarding their contributions to the biological properties of the extract, especially antimicrobial and antibiofilm activities. Additionally, this accurate quantification method provided insight into understanding the chemical profile of *L. capitata*. Also, it could be employed in the quantitative measurement of both biomarkers in natural products containing this plant and in its quality control.

## 4. Materials and Methods

### 4.1. Plant Material

The entire *Launaea capitata* (Spreng.) Dandy plant was used in the current study. The detailed procedures of the plant authentication, drying, powdering, and extraction were previously reported by our research group [27].

### 4.2. Biomarker Compounds

Compounds (**1** and **2**) utilized in this study were extracted from the *n*-hexane fraction of the total methanol extract of the entire *L. capitata* plant. Their identification followed the methodology described in a prior publication by our research team [27]. Their purity was confirmed by ^1^H and ^13^C NMR spectral analysis (see Supplementary Figures S1–S4).

### 4.3. Bacteria and Culture Media

A total of nineteen *K. pneumoniae* isolates were obtained from Tanta University Hospital. Biochemical tests [61], as well as the matrix-assisted laser desorption/ionization-time of flight mass spectrometry MALDI-TOF MS, bioMérieux (Marcy l’Etoile, France), were utilized to identify the isolates. The used culture media were purchased from Merck (Darmstadt, Germany).

### 4.4. In Vitro Studies

#### 4.4.1. Susceptibility Testing 

The sensitivity to the studied chemicals was assessed by agar diffusion assay using concentrations of 2000 μg/mL as previously described [62] in Muller-Hinton agar plates. The tested minimum inhibitory concentrations (MICs) of compounds were detected by broth dilution [63] employing ciprofloxacin and dimethyl sulfoxide as positive and negative controls, as previously described. The MICs were the minimum concentrations that hindered the growth [63].

#### 4.4.2. Inhibition of Biofilm Formation

As previously described [63], the phenotypic investigation of the biofilm inhibition was performed using crystal violet assay in 96-well microdilution plates. The free-floating bacteria were removed from the overnight grown bacterial suspensions in microdilution plates. After rinsing the wells, 200 µL of crystal violet (0.1%) solution was utilized to stain the attached bacteria for 20 min. After that, the wells were washed thrice and left to dehydrate. The remaining stain bound to the cells was solubilized with glacial acetic acid (200 µL). Finally, the absorbance was recorded at 490 nm using a Sunrise ELISA reader (Grödig, Austria) [63]. 

#### 4.4.3. qRT-PCR

The expression of the *lux*S, *mrk*A, *wzm*, and *wbb*m genes was quantified in *K. pneumoniae* isolates by qRT-PCR. The 1*6SrRNA* gene was utilized as a reference gene. In brief, the biofilm-forming bacterial isolates were incubated with 0.5 MIC tested compounds. Then, the total RNA was extracted by a Qiagen RNA extraction kit (Hilden, Germany), and then cDNA was formed by a Qiagen cDNA synthesis kit (Hilden, Germany). Finally, qRT-PCR was run [11]. The primers are displayed in Supplementary Table S1.

### 4.5. In Vivo Explorations against K. pneumoniae

#### 4.5.1. Experimental Procedures

We obtained forty mice from Cairo University, Egypt, which were preserved and fed according to the outlined standard conditions [64]. The protocol was endorsed by the Research Ethics Committee of The Faculty of Pharmacy, Tanta University, Tanta, Egypt (TP/RE/2/24 p-02). The antibacterial action of **1** against *K. pneumoniae* bacteria was revealed in vivo. After injecting the *K. pneumoniae* suspension (10^7^ CFU)/mL) twice through two days, intraperitoneally, into the thirty mice, the mice were allocated into four groups. Group I was not infected and was administered 0.9% normal saline (normal control). Groups II, III, and IV were injected with *K. pneumoniae* and administered 0.9% normal saline (positive control), compound **1**, and ciprofloxacin, respectively, each day [62]. After ten days, the mice were anesthetized, their liver and spleen tissues were acquired, and the bacterial count was revealed in the tissues homogenate.

#### 4.5.2. Histopathology and Immunohistochemistry (IHC)

Following the formerly described method [62], the liver and spleen were stained with hematoxylin and eosin (H&E) stain. Regarding the immunohistochemical analysis of the studied tissues, tumor necrosis factor-alpha (TNF-*α*) monoclonal antibodies were employed to stain the tissues. After staining, the tissues were investigated using a light microscope.

#### 4.5.3. ELISA 

Interleukin one beta (IL-1*β*) and interleukin six (IL-6) were quantified in the tissues of the different groups by an ELISA kit from Abcam Co. (Waltham, MA, USA).

### 4.6. Statistics

The potential statistical significance of the tested compounds was judged by ANOVA at *p* < 0.05 by GraphPad Prism 8.0 software (San Diego, CA, USA). The experimental analyses were employed thrice as the mean ± standard deviation (SD).

### 4.7. HPLC-PDA Determination of **1** and **2** in the Total Extract of Launaea capitata

#### 4.7.1. Mobile Phase

Methanol (HPLC grade, >99.9%) and acetonitrile (HPLC grade, >99.9%) were obtained from Sigma-Aldrich (St. Louis, MO, USA). Filtered double dist. H_2_O was utilized during the analysis. 

#### 4.7.2. Preparation of Standard Solution

The stock solutions of compounds **1** and **2** (100 μg/mL) were prepared by the dissolution of 10 mg in a final volume of 100 mL of methanol. The stock solutions were further diluted using the same solvent to get working solutions. When stored at 4 °C, the resultant solutions remained stable for at least two weeks.

#### 4.7.3. Software and Instrument 

The study employed a Dionex UltiMate 3000 HPLC system (Thermo Scientific™, Dionex™, Sunnyvale, CA, USA). The instrument was set up with a quaternary pump (LPG-3400SD), an autosampler (WPS-3000TSL), a column thermostat (TCC-3000SD), and a photodiode array detector (PDA). The data collecting and processing were performed using Chromeleon 7 software. The investigation employed a Hypersil BDS C18 column (250 mm × 4.6 mm i.d.) and the particle size was 5 μm.

#### 4.7.4. Chromatographic Conditions

The determination of **1** and **2** compounds was conducted utilizing an RP-C18 column (Hypersil BDS, 5 μm particle size, 250 mm × 4.6 mm i.d.) with a mobile phase composed of a mixture of methanol and double dist. H_2_O (50:50% *v/v*). The process of isocratic elution was carried out at a flow rate of 1.0 mL/min and an injection volume of 30.0 μL. The photodiode array (PDA) detector was configured to operate at a wavelength of 284 nm in order to analyze and quantify the substances under investigation. Prior to the examination, it was vital to condition the column for a duration of 20 min.

#### 4.7.5. Construction of Calibration Curves

Different known aliquots of the standard solution for both **1** and **2** compounds (100.0 μg/mL) were transferred into volumetric flasks (5.0 mL) and filled with mobile phase up to the mark. This resulted in solutions with concentrations ranging from 5.0–40.0 µg/mL for **1** and 1.0–40.0 µg/mL for **2**. Under the optimal chromatographic conditions, a volume of 30.0 µL from each solution was injected in triplicate. The calibration curves were generated by graphing the concentration of each marker compound in µg/mL against the peak area, and subsequently deriving the regression equations. The concentration of each compound in the total extract was determined using the corresponding regression equation.

## 5. Conclusions

Herein, we elucidated the potential antibacterial, antibiofilm, and anti-quorum-sensing actions of the methanol extract of the entire *L. capitata* plant alongside two main polyacetylene glycosides (**1** and **2**) isolated from this plant material on *K. pneumoniae* clinical isolates. qRT-PCR showed the potential antibiofilm action of (3*S*,6*E*,12*E*)-6,12-tetradecadiene-8,10-diyne-1-ol 3-*O*-*β*-D-glucopyranoside (**1**) at the biofilm and QS gene expression level. The in vivo experiment revealed the effectiveness of **1** in the animal model by decreasing the bacterial burden and the inflammatory mediators in addition to improving the histological attributes of the liver and spleen. Thus, **1** could have a potential role in our fight against evolving bacterial resistance. A precise HPLC-PDA quantification method was developed, which offered a valuable tool for quantifying these biomarkers in herbal products containing this plant and for quality control purposes. The significant presence of compound **1** in the plant extract at a substantial level suggests its potential contributions to the traditional use of this plant in the treatment of fever and infected wounds, particularly due to antimicrobial and antibiofilm activities. This traditional knowledge aligns with modern scientific exploration, highlighting the value of integrating ethnobotanical practices with contemporary research to validate and understand the medicinal properties of such plants. Furthermore, the accurate HPLC-PDA quantification method not only serves as a valuable tool for measuring biomarkers **1** and **2** in natural products containing *L. capitata* but also plays a role in quality control purposes of the plant.

## Figures and Tables

**Table 1:** The MIC values of total *L. capitata* extract, **1**, **2**, and ciprofloxacin against *K. pneumoniae* clinical isolates.

*K. pneumoniae* Isolates	MIC (µg/mL) ^1^			
	1	2	Total Extract	Ciprofloxacin
K1	16	1024	512	0.125
K2	128	1024	512	0.25
K3	32	1024	1024	0.25
K4	128	2048	512	0.25
K5	32	2048	1024	0.25
K6	32	2048	1024	0.125
K7	128	2048	512	0.25
K8	32	1024	512	0.25
K9	64	1024	512	0.25
K10	128	2048	1024	0.125
K11	32	2048	512	0.25
K12	32	1024	512	0.25
K13	32	1024	512	0.125
K14	64	2048	1024	0.25
K15	64	2048	1024	0.125
K16	32	1024	512	0.125
K17	16	1024	1024	0.25
K18	64	1024	512	0.125
K19	128	2048	512	0.125

^1^ Minimum inhibitory concentration (MIC) measured with the broth dilution method.

**Table 2:** Impact of the total extract, **1,** and **2** on the biofilm of *K. pneumoniae* isolates.

Capacity to Form Biofilm	Isolate Count			
	Before Treatment	After Treatment		
		1	2	Total Extract
No	2	4	2	2
Weak	1	7	2	4
Moderate	7	3	7	7
Strong	9	5	8	6

**Table 3:** Analytical performance data for the proposed method.

Parameters	1	2
Linearity range (µg/mL)	5.0–40.0	1.0–40.0
Intercept (a)	−0.0954	0.7381
Slope (b)	0.0245	0.1572
Correlation coefficient (r)	0.9995	0.9998
S.D. of the residuals, S_y/x_	0.0085	0.0185
S.D. of the intercept, S_a_	0.0038	0.0045
S.D. of the slope, S_b_	0.0003	0.0006
Percentage relative standard deviation, % RSD	2.485	3.092
Percentage relative error, % Error	1.116	1.256
Limit of detection, LOD (µg/mL) ^1^	0.518	0.096
Limit of Quantitation, LOQ (µg/mL) ^2^	1.568	0.289

^1^ LOD = 3.3 Sa/b, ^2^ LOQ = 10 Sa/b, where Sa = standard deviation of the intercept and b = slope.

**Table 4:** Utilization of the proposed method for quantifying the analyzed compounds in their pure forms.

Parameter	1			2		
Concentrations	Amount taken (µg/mL)	Amount found (µg/mL)	Percentage found ^1^	Amount taken (µg/mL)	Amount found (µg/mL)	Percentage found ^1^
	5.0	5.12	102.37	1.0	1.03	102.99
	10.0	10.26	102.61	5.0	4.78	95.53
	15.0	14.91	99.40	10.0	9.94	99.36
	20.0	19.48	97.41	15.0	15.02	100.17
	40.0	40.18	100.45	20.0	20.39	101.97
				40.0	39.83	99.59
Mean “X¯”			100.45			99.51
±S.D			2.50			3.08
% RSD			2.485			3.092
% Error			1.116			1.256

^1^ Each result is the average of three separate determinations.

## Data Availability

The data supporting the findings of this research are available within the Supplementary Materials.

## Supplementary Materials

The following supporting information can be downloaded at: https://www.mdpi.com/article/10.3390/ph17091214/s1, Figures S1 and S2: ^1^H and ^13^C NMR spectra of compound **1**; Figures S3 and S4: ^1^H and ^13^C NMR spectra of compound **2**; Table S1: Primer sequences.
