# Supplementary material for: HPLC-PDA Analysis of Polyacetylene Glucosides from Launaea capitata and Their Antibacterial and Antibiofilm Properties against Klebsiella pneumoniae
Source: Pharmaceuticals (Basel). 2024 Sep 15;17(9):1214. doi: 10.3390/ph17091214 (PMC11434657; doi:10.3390/ph17091214)
Supplement: Supplementary file 1 [file pharmaceuticals-17-01214-s001.zip › pharmaceuticals-3200323-supplementary.pdf]

Supplementary materials

HPLC-PDA Analysis of Polyacetylene Glucosides from *Launaea capitata* and their Antibacterial and Antibiofilm Properties against *Klebsiella pneumoniae*

Tariq M. Aljarba [1], Fatma M. Abdel Bar [1, 2] *, Asmaa E. Sherif [1,2], Engy Elekhnawy [3], Galal Magdy [4,5], and Reham M. Samra [2]

1 Department of Pharmacognosy, College of Pharmacy, Prince Sattam Bin Abdulaziz University, Al-Kharj, 11942, Saudi Arabia; t.aljarba@psau.edu.sa; f.abdelbar@psau.edu.sa; ae.sherif@psau.edu.sa

2 Department of Pharmacognosy, Faculty of Pharmacy, Mansoura University, Mansoura 35516, Egypt; rehamsamra@mans.edu.eg

3 Pharmaceutical Microbiology Department, Faculty of Pharmacy, Tanta University, Tanta 31527, Egypt; engy.ali@pharm.tanta.edu.eg

4 Pharmaceutical Analytical Chemistry Department, Faculty of Pharmacy, Kafrelsheikh University, Kafrelsheikh 33511, Egypt; galal_magdy@pharm.kfs.edu.eg

5 Department of Pharmaceutical Analytical Chemistry, Faculty of Pharmacy, Mansoura National University, Gamasa, 7731168, Egypt

* Correspondence: Fatma M. Abdel Bar, E-Mail: f.abdelbar@psau.edu.sa; Tel.: +966-54540-3617

Table of Contents **Page**

Figure S1. ^{1}H NMR spectrum of compound **1.** 3

Figure S2. ^{13}C NMR spectrum of compound **1**. 4

Figure S3. ^{1}H NMR spectrum of compound **2**. 10

Figure S4. ^{13}C NMR spectrum of compound **2**. 11

Table S1. Primer sequences 31

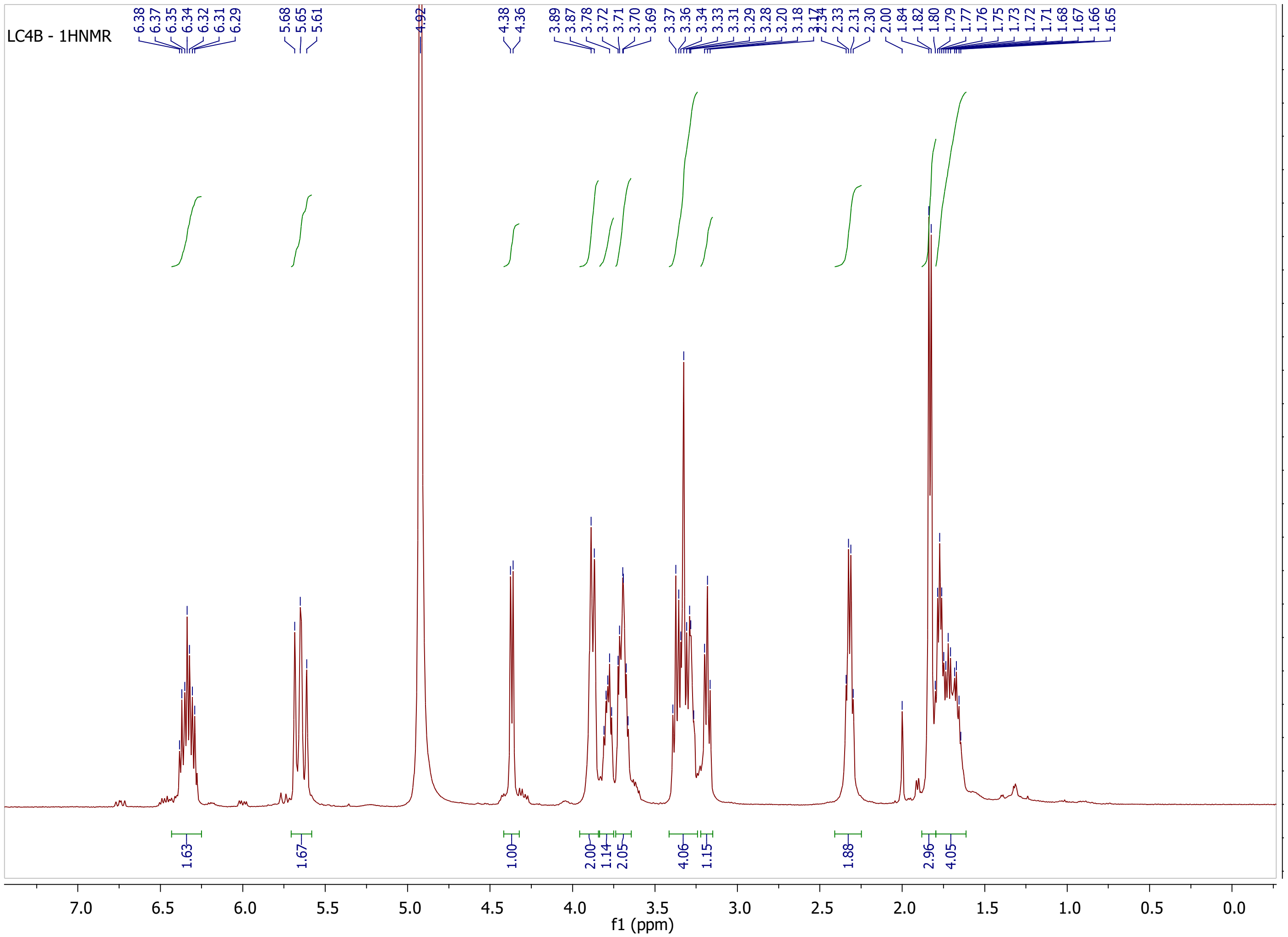

Figure S1. ^{1}H NMR spectrum of compound **1** (CD_3OD, 500 MHz).

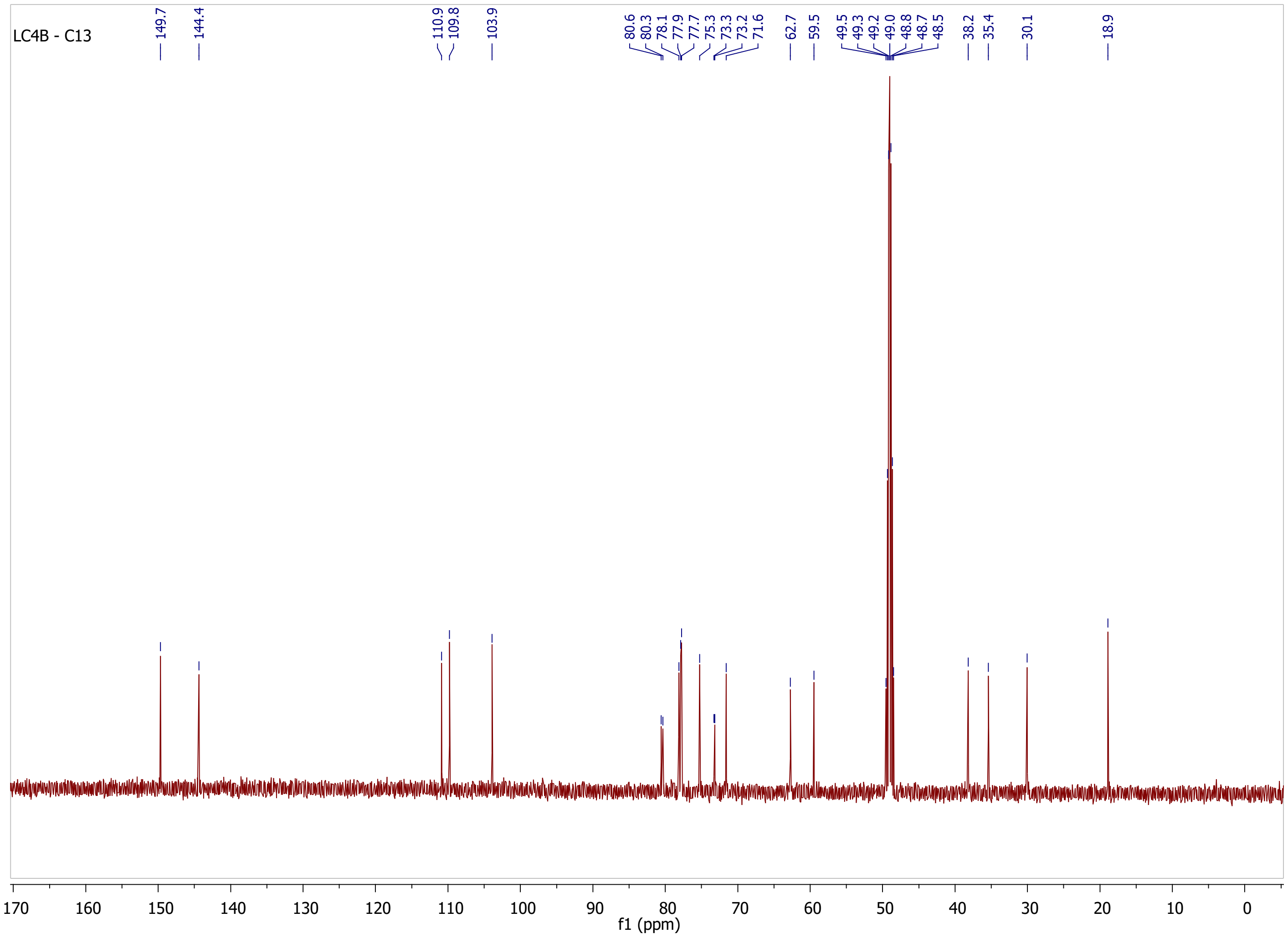

Figure S2. ^{13}C NMR spectrum of compound **1** (CD_3OD, 125 MHz).

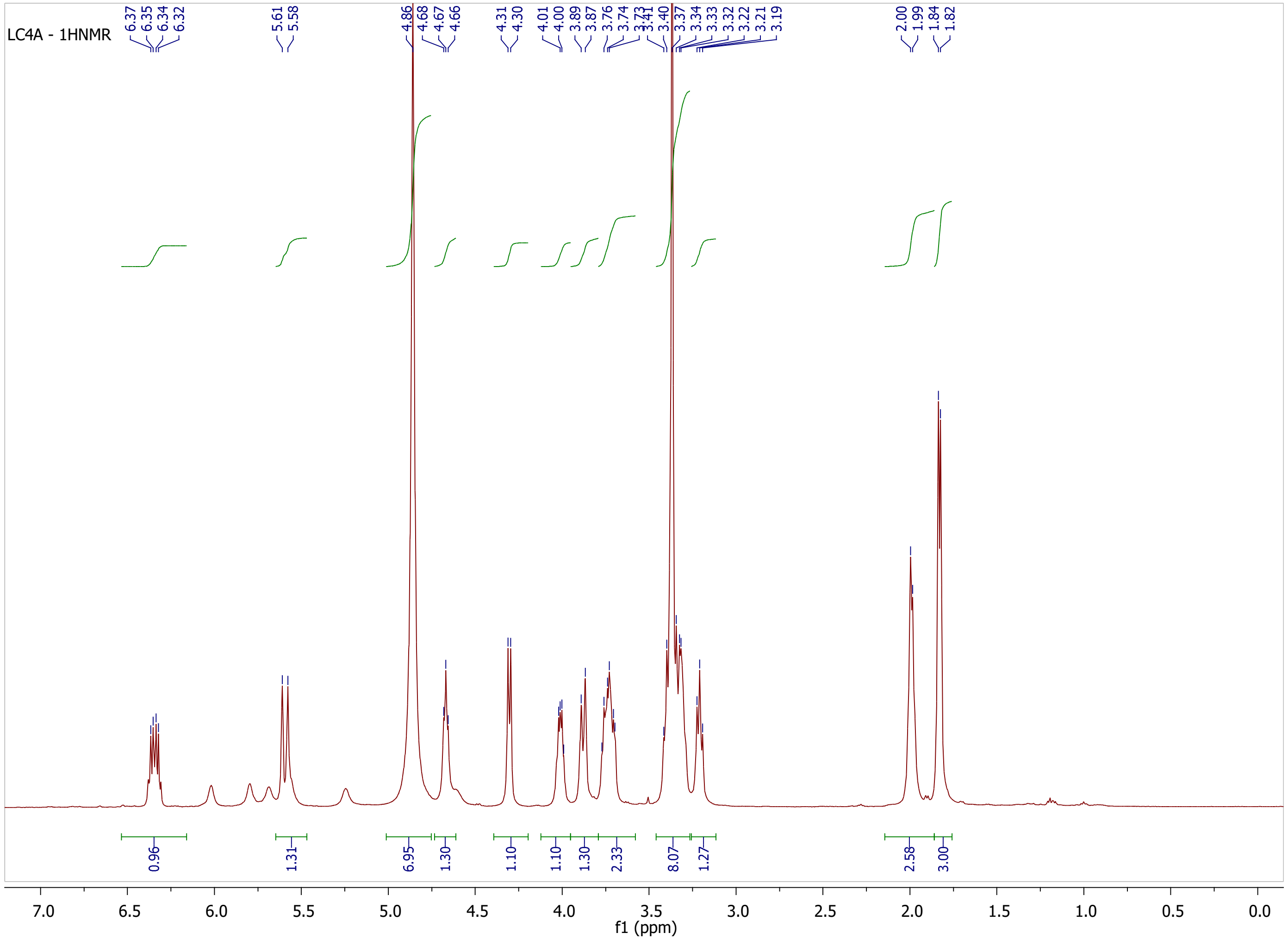

Figure S3. ^{1}H NMR spectrum of compound **2** (CD_3OD, 500 MHz).

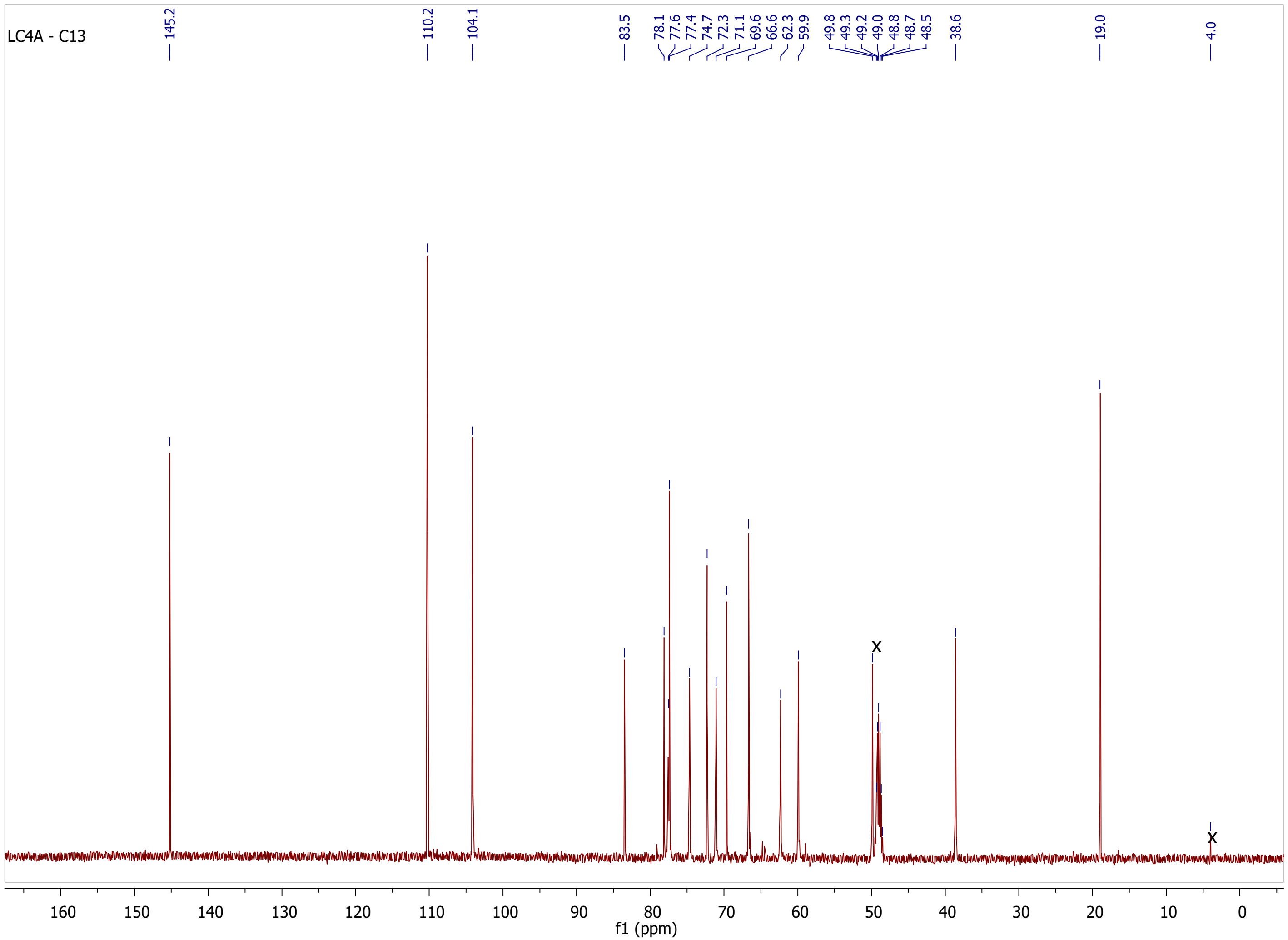

Figure S4. ^{13}C NMR spectrum of compound **2** (CD_3OD, 125 MHz).

Table S1. Primer sequences

Primer	Sequence
luxs	F: 5′- ACGCCATTACCGTTAAGATG -3′
	R: 5′- TGTCGTCAGCTCGTGTTATG -3′
mrkA	F: 5′- AGCGATGCGAACGTTTACCTGTCTC -3′
	R: 5′- CGTCATCCTGTTTAGTGCCATCAGC -3′
wzm	F: 5′- CTATCGAAGACGTATCCTTTAC -3′
	R: 5′- ATATTCTCACGCCCGGTAAG -3′
wbbM	F: 5′- TTATCAGGCTGCCATTGCCAT -3′
	R: 5′- CAGCTATATGCCCAATAACGC -3′
16srRNA	F: 5′- CTGTGGATGCTCAAGGACTAC -3′
	R: 5′- ATCCCCACCTTCCTCCAGTT -3′
